# Supplementary material for: Locating ligand binding sites in G-protein coupled receptors using combined information from docking and sequence conservation
Source: PeerJ. 2021 Sep 24;9:e12219. doi: 10.7717/peerj.12219 (PMC8475542; doi:10.7717/peerj.12219)
Supplement: Supplemental Information 2 [file peerj-09-12219-s002.docx]

**TITLE: Locating ligand binding sites in G-protein coupled receptors using combined information from docking and sequence conservation**

Authors: Ashley R. Vidad, Stephen Macaspac, & Ho-Leung Ng

SUPPLEMENTARY INFORMATION

Figure legends

**Figure S1. Four experimentally verified GPER ligands.**

**Figure S2. ConSurf multiple sequence alignment for GPER.**

**Figure S3. Consurf surfaces for A2A adenosine and β2 adrenergic receptors.**

**Figure S4. CASTp geometric ligand binding site predictions for A2A adenosine and β2 adrenergic receptors.**

**Figure S5. Overlay of GPER homology model with the crystal structure of the** **CCR5 chemokine receptor.** GPER is green. CCR5 receptor is blue. E2 poses are in blue. The top of the figure corresponds to the extracellular

**Figure S6. Ramachandran plot of the GPER homology model.** Calculated by Chimera.

**Figure S7. ERRAT analysis of the GPER homology model.**

**Figure S8. Predicted ligand binding pockets in GPER.** A) Extracellular perspective of GPER showing amino acids predicted to contact ligands E2 (maroon), G1 (cyan), G15 (green), and tamoxifen (violet). Residues colored orange are predicted to contact one or more ligands with darker hue indicating interaction with multiple ligands. B) Predicted binding pocket viewed from a 90° rotation.

**Figure S9. Comparison of proposed ligand binding sites.** Comparison of the ConDock ligand binding site (red) with that proposed by Mendez-Luna *et al.* (blue). Residues found in both sites are colored violet.

**Table S1. ConDockSite predicted ligand binding sites for A2A adenosine and β2 adrenergic receptors using crystal structures.**

**Table S2. ConDockSite predicted ligand binding sites for eight GPCRs using homology models.**

**Table S3. Scores for predicted binding sites and poses for GPER ligands.**

**Figure S1. Four experimentally verified GPER ligands**


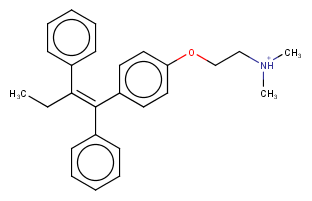

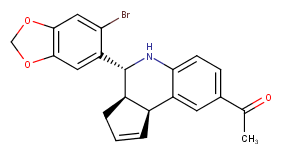


17β-estradiol

tamoxifen

G1


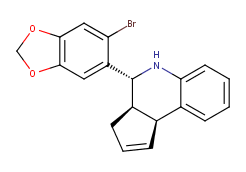

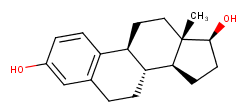


G15

**Figure S2. ConSurf multiple sequence alignment for GPER.**

**Figure S3. Consurf surfaces for A2A adenosine and β2 adrenergic receptors.**


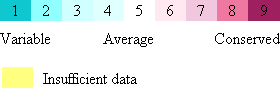

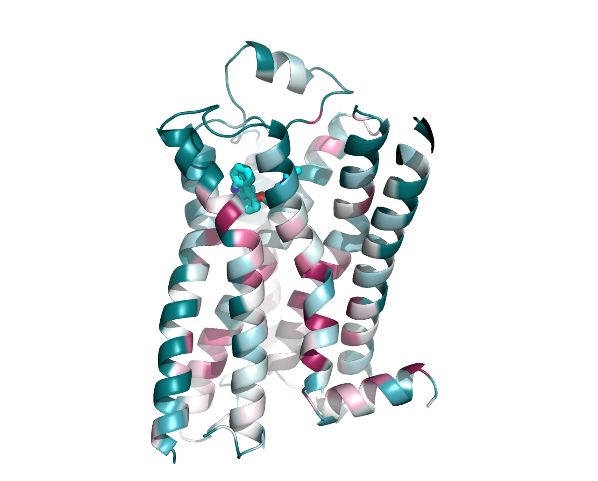

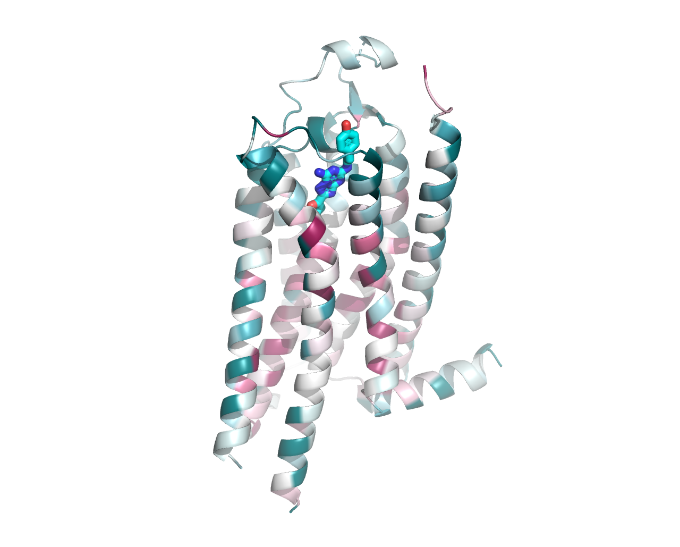
 A2A adenosine β2 adrenergic

**Figure S4. CASTp geometric ligand binding site predictions for A2A adenosine and β2 adrenergic receptors.** Interacting residues are shown in blue.


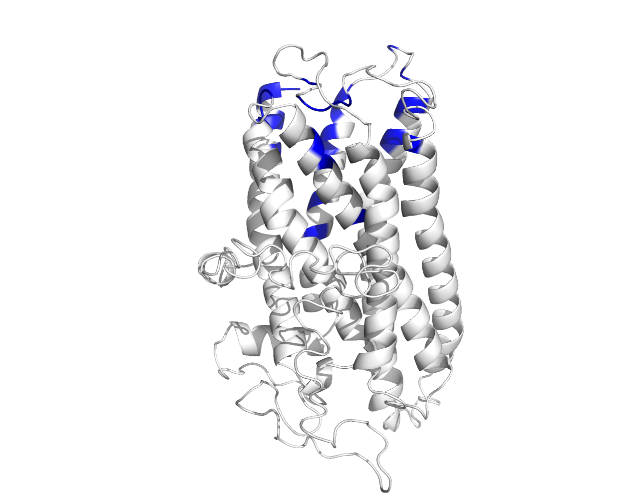

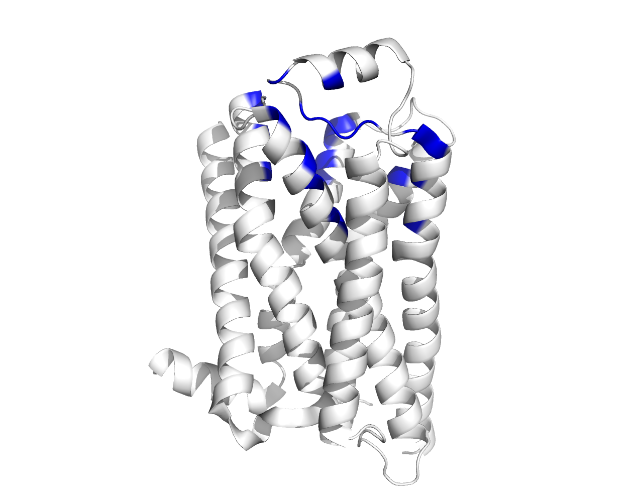
 A2A adenosine β2 adrenergic

**Figure S5. Overlay of GPER homology model with the crystal structure of the** **CCR5 chemokine receptor.** GPER is green. CCR5 receptor is blue. E2 poses are in blue. The top of the figure corresponds to the extracellular face.


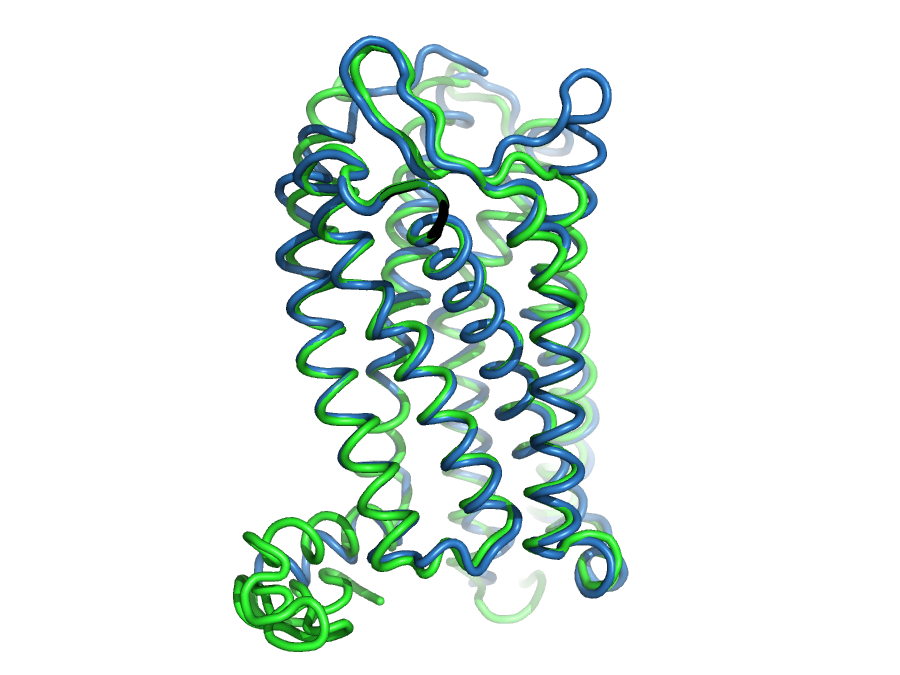


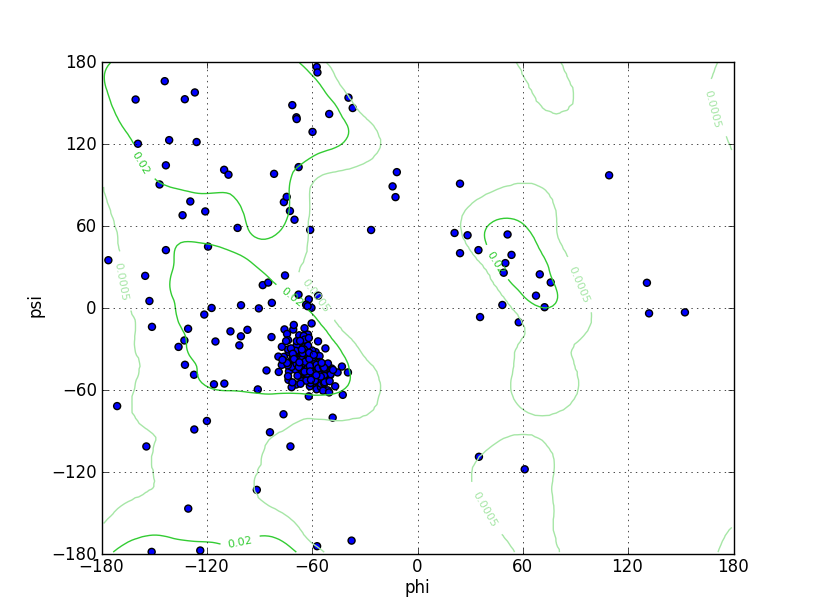


**Figure S6. Ramachandran plot of the GPER homology model.** Calculated by Chimera.

**Figure S7. ERRAT analysis of the GPER homology model.**


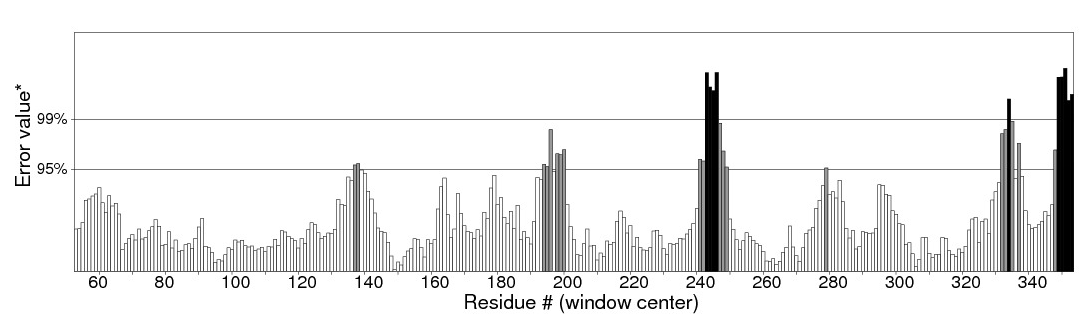


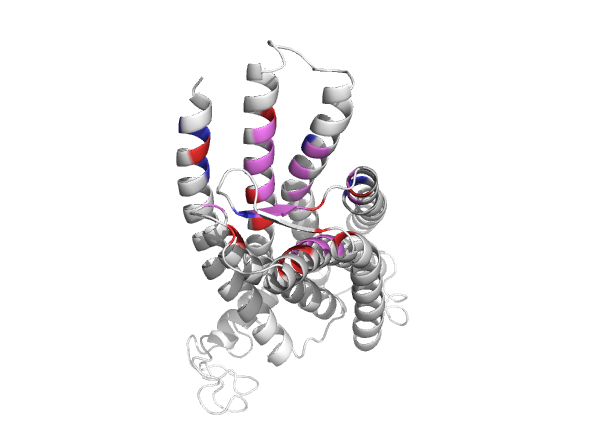


A)

B)


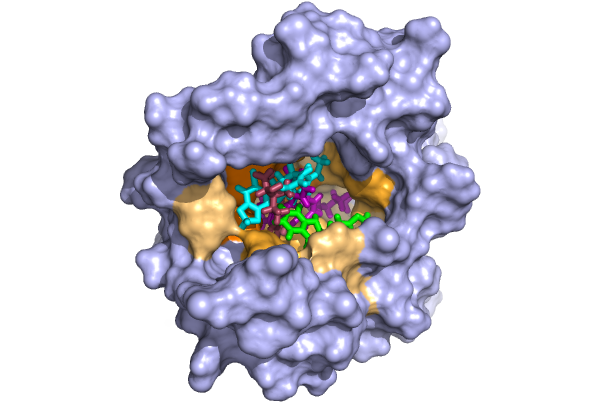

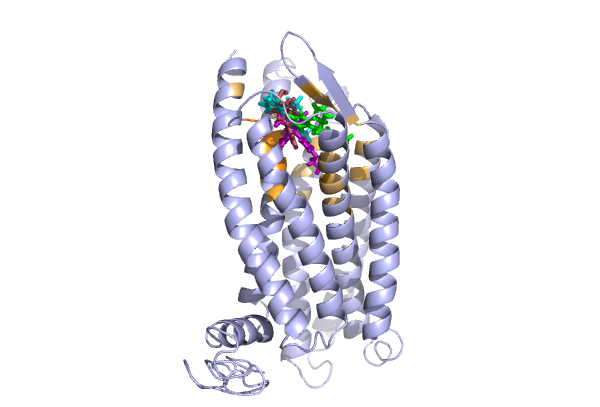


**Figure S8. Predicted ligand binding pockets in GPER.** A) Extracellular perspective of GPER showing amino acids predicted to contact ligands E2 (maroon), G1 (cyan), G15 (green), and tamoxifen (violet). Residues colored orange are predicted to contact one or more ligands with darker hue indicating interaction with multiple ligands. B) Predicted binding pocket viewed from a 90° rotation.

**Figure S9. Comparison of proposed ligand binding sites.** Comparison of the ConDockSite ligand binding site (red) with that proposed by Mendez-Luna *et al* (2015)*.* (blue). Residues found in both sites are colored violet.

**Table S1. ConDockSite predicted ligand binding sites for A2A adenosine and β2 adrenergic receptors using crystal structures.**

| **GPCR** | **Ligand** | **Distance between ConDockSite and crystal structure poses (Å)** | **ConSurf conservation score** | **SwissDock energy Score** | **ConDockSite score** |
| --- | --- | --- | --- | --- | --- |
| A2A adenosine | adenosine  (agonist) | 0.4 | 0.86 | -1093.0 | -940.0 |
| A2A adenosine | ZM241385  (inverse agonist) | 0.4 | 0.86 | -1103.5 | -949.0 |
| β2 adrenergic | epinephrine  (agonist) | 0.4 | 0.87 | -1177.3 | -1024.3 |
| β2 adrenergic | carazolol  (inverse agonist) | 1.0 | 0.78 | -1170.7 | -913.1 |

**Table S2. ConDockSite-predicted ligand binding sites for eight GPCR-ligand complexes using homology models.**

| **GPCR** | **Ligand** | **Cα rmsd between homology model and crystal structure (Å)** | **Distance between ConDockSite and crystal structure ligand poses (Å)** |
| --- | --- | --- | --- |
| A2A adenosine | ZM241385 | 2.1 (1.6) | 4.2 (5.4) |
| β2 adrenergic | carazolol | 1.1 (1.8) | 1.8 (2.3) |
| Mu opioid | BU72 | 1.8 | 3.2 |
| Serotonin 5HT2B | methysergide | 1.9 | 7.0 |
| Dopamine D2 | haloperidol | 1.6 | 4.3 |
| Dopamine D2 | risperidone | 1.8 | 2.4 |
| Ghrelin | Compound 21 | 1.7 | 3.1 |
| Histamine H1 | doxepin | 1.5 | > 10 |
| Muscarinic M1 | tiotropium | 2.1 | > 10 |

**Table S3. Scores for predicted binding sites and poses for GPER ligands.**

| **Ligand** | **ConSurf conservation score** | **SwissDock energy score** | **Combined ConDock score** |
| --- | --- | --- | --- |
| E2, pose 1 | 0.84 | -964.9 | -813.2 |
| E2, pose 2 | 0.80 | -967.9 | -774.3 |
| G1 | 0.85 | -970.7 | -825.1 |
| G15 | 0.8 | -974.6 | -779.7 |
| Tamoxifen, pose 1 | 0.81 | -947.9 | -763.6 |
| Tamoxifen, pose 2 | 0.81 | -947.5 | -763.6 |
